# Poor incomes and economic hardships among commercial motorcycle drivers (boda-boda) are associated with accidents and injuries in Gulu Municipality, Northern Uganda: a cross-sectional study

**DOI:** 10.11604/pamj.2022.41.274.31302

**Published:** 2022-04-05

**Authors:** David Lagoro Kitara, Eric Nzirakaindi Ikoona

**Affiliations:** 1 Department of Global Health and Populations, Harvard University, Harvard T.H. Chan School of Public Health, Boston, Massachusetts, United States of America,; 2 Department of Surgery, Gulu University, Faculty of Medicine, Gulu City, Uganda,; 3 International Center for AIDS Care and Treatment Programs (ICAP) at the University of Columbia, Sierra Leone

**Keywords:** Boda-boda accidents, boda-boda drivers, Gulu Municipality, low incomes, behaviours, injuries

## Abstract

**Introduction:**

injuries in commercial motorcycle drivers (boda-boda) are the second-commonest reason for trauma-related admission to the Gulu Regional Referral Hospital. Most causes of boda-boda accidents and injuries were related to the behaviors of drivers, passengers, and pedestrians. The purpose of this study was to determine factors associated with boda-boda drivers, accidents, and boda-boda accident victims in Gulu Municipality, Northern Uganda.

**Methods:**

two cross-sectional studies were conducted at intervals of six months between July and December 2015. Two hundred boda-boda drivers from Gulu Municipality and fifty-seven victims of boda-boda accidents admitted to the Gulu Regional Referral Hospital were recruited for this study. A pre-tested questionnaire with Cronbach´s alpha (internal validity) α=072 was used for data collection. This study was approved by a local Institutional Review Board (IRB) and STATA version 14.1 was used for statistical analysis. A p-value less than 0.05 was considered statistically significant.

**Results:**

factors associated with boda-boda accidents in Gulu Municipality were boda-boda drivers from Pece division (AoR=7.290, 95% CI: 2.162-24.580; p<0.001) and those with low monthly incomes less than UGX400,000/= equal to USD$100 (AoR=0.154, 95% CI: 0.031-0.766; p<0.05). Drivers with monthly incomes higher than UGX400,000/= were least likely involved in boda-boda accidents (AoR=0.104, 95% CI: 0.038-0.281; p<0.001). Work experience, prior road safety training, age, wearing a helmet and protective clothing, levels of education, and knowledge on road safety regulations did not significantly affect the outcome. Most victims of boda-boda accidents were passengers and pedestrians from villages outside Gulu Municipality (AoR=8.808, 95% CI: 3.190-24.329; p<0.001) and sustained minor injuries.

**Conclusion:**

boda-boda accidents in Gulu Municipality are problematic, drivers from the Pece division and those with low monthly incomes were more involved. Most victims of boda-boda accidents were passengers and pedestrians from villages outside Gulu Municipality. This study suggests that boda-boda drivers should be engaged in other income-generating activities, as some divisions in Gulu Municipality do not generate the required resources to meet their needs.

## Introduction

Boda-boda as described in the English Oxford Dictionary is a type of commercial motorcycle or bicycle with a space for a passenger or carrying goods, and often used as a commercial motorcycle [1]. Its name was derived from lucrative trade between the borders of Uganda and Kenya in East Africa [1]. In the current use, they are key and agile transport systems which are used in most urban and rural areas of Uganda for quick arrivals, beating vehicular traffic jams, transfer of patients from and to hospitals, and easy reach to unnavigable places [2]. Most boda-boda motorcycles have low engine capacities (50-100cc) [3], are economically expedient because they consume little fuel (gas), and are technically easy to operate [2,3]. Despite the advantages of the boda-boda transport system, these commercial motorcycles are involved in a substantial number of road traffic accidents and many associated injuries in Uganda [4-7]. Boda-boda drivers are the second-largest road user category involved in road traffic injuries (RTIs) in Uganda [3] and according to a Ugandan road safety performance review report, road fatalities involving boda-boda drivers doubled between 2011 and 2015 (from 570 deaths to 1170 deaths) [8]. Mulago, the National Referral Hospital (MNRH) receives between 10 and 20 cases of boda-boda related RTIs daily and spends more than 60% of its annual surgery budget on treating trauma from boda-boda injuries [7,9]. Poor riding behaviors for example over-speeding, unsafe overtaking, and low use of protective gears have been blamed for the increased crashes and injury risk among boda-boda drivers [6,7,10,11]. Studies in several countries found factors that predisposed commercial motorcycles to crashes for example age, ethnicity, levels of education, motorcycle license insurance status, self-reported alcohol consumption in the twelve hours preceding the crash, years of on-road riding experience, kilometers traveled on the specific motorcycle, posted speed limit, weather conditions [12-17], motorcycle engine sizes, time of the occurrence [18], motorcycle ownership, over-speeding and risk-taking behaviors [19]. Thus, boda-boda is one of the major causes and agents of road traffic injuries in Uganda [6,20] and causes significant economic burdens to Uganda and individuals as the treatment cost of each person is estimated between USD300-369 [6,20].

Captivatingly, the number of road traffic accidents attributable to boda-boda drivers has been increasing annually in Uganda and particularly in the urban areas [20]. This was similarly observed in a Ugandan police report which showed that boda-boda was the leading cause of accident scene fatalities in Kampala, Uganda [21]. Moreover, a Ugandan injury report of 2010 showed that 17% of 2,954 people killed in road traffic injuries were operators or passengers of two or three-wheeled vehicles, primarily commercial motorcycles (boda-bodas) [22]. Some authors have reasoned that boda-boda commercial motorcycles were filling major gaps in the Ugandan transportation system and that they operated were more conventional transportation services were uneconomical or were physically impossible [2,3,23]. They argued that it was important to view boda-boda commercial motorcycles as lucrative, agile, cheap, affordable and that it was likely to remain one of the most important determinants in the transport sector development in Uganda in the many years to come [2,3,23]. Similarly, boda-boda accidents in Northern Uganda are problematic, they contribute to 24.1% of all physical trauma treated in Gulu Regional Referral Hospital, thus contributing to quick depletion of hospital resources, increased hospital bed occupancy rates, and more disabilities among the population of Northern Uganda, an area which is emerging from a 20-year-old civil war [5,24]. Authors have suggested that there were needs to gather as much information on boda-boda drivers and accidents so that predictable efforts could be made to regulate and make it safer for other road users. The need to learn more about boda-boda drivers is part of a process to support all efforts to reduce the high prevalence of road traffic accidents and injuries in Gulu Municipality. The objective of this study was to determine factors associated with boda-boda drivers, accidents, and victims of boda-boda accidents in Gulu Municipality of Northern Uganda.

## Methods

**Study design:** two cross-sectional studies were conducted at six months´ intervals on two hundred boda-boda drivers in Gulu Municipality between July and December 2015. In the same period, fifty-seven victims of boda-boda accidents were recruited at Gulu Regional Referral Hospital.

**Study settings:** this study was conducted in Gulu Municipality and Gulu Regional Referral Hospital. Gulu Municipality is one of the major towns in Northern Uganda, approximately 343 kilometers north of Kampala, the capital city of Uganda. It is reported that it is one of the fastest-growing urban centers in Uganda. It is also a major urban center in Northern Uganda and a gateway to lucrative businesses in South Sudan. The municipality is situated on the Great North Road which connects Cape Town in South Africa to Cairo in Egypt (North Africa). Data collection was undertaken at the boda-boda road-side stages located along major routes in the four divisions of Gulu Municipality (Bardege, Layibi, Pece, and Laroo). Each boda-boda stage was strategically located and was well organized with a management structure and a stage leader. The function of the leader was to regulate the operations of members and organize collective actions in times of need. On the other hand, Gulu Regional Referral Hospital is the main public hospital and a teaching hospital for Gulu University Medical School. It is located at the center of Gulu Municipality and offers free emergency medical and surgical services to victims of accidents. The hospital has an accident and emergency department (entry point), surgical wards (male and female wards), and operating theatres for the management of trauma cases. Hospital data for this study were collected from the surgical units of Gulu Regional Referral Hospital.

**Study population:** the study population was boda-boda drivers from the four divisions of Gulu Municipality and victims of boda-boda accidents who were treated at the surgical units of Gulu Regional Referral Hospital (accident and emergency, operating theatres, and surgical wards).

**Selection of participants:** boda-boda drivers were selected consecutively from boda-boda stages scattered along major routes in the four divisions of Gulu Municipality. Similarly, victims of boda-boda accidents were consecutively selected from the Surgical Units of Gulu Regional Referral Hospital from July to December 2015.

### Selection criteria

**Inclusion and exclusion criteria for drivers:** for boda-boda drivers, three conditions had to be fulfilled before recruitment; 1) the driver was a registered member of a stage; 2) had been operating in Gulu Municipality for three or more months consecutively before this study; 3) had written informed consent. Those without the three requirements were excluded from the study.

**Inclusion and exclusion criteria for victims of boda-boda accidents:** victims of the boda-boda accidents were recruited on condition that they had; i) a medical form five from Gulu Regional Referral Hospital with evidence that they had sustained boda-boda injuries in Gulu Municipality; ii) police confirmation; iii) written informed consent, and; (iv) the victim was conscious and capable of taking part in interviews. Those without the four requirements for recruitment were excluded from the study.

**A sampling of participants:** boda-boda drivers and victims of boda-boda accidents were sampled consecutively from their work stations and Gulu Regional Referral Hospital Surgical Units respectively. We consecutively recruited drivers (targeting them at roadside stages in all the four divisions of Gulu Municipality) to the study until the required sample size was attained. This method of sample size determination had been previously used in several studies conducted on boda-boda drivers in Uganda [2,3,5,21]. Similarly, hospital data was obtained by recruiting victims of boda-boda accidents consecutively from the surgical units of Gulu Regional Referral Hospital from July to December 2015.

**Sample size estimation:** we used the total number of registered boda-boda drivers in Gulu Municipality as the total population for computing the sample size for our study population. Municipal authorities and the police reported six hundred (600) as the number of registered boda-boda drivers in Gulu Municipality [25]. A previous study conducted in the same setting informed the authors that 24.1% of injuries treated at Gulu Regional Referral Hospital were due to boda-boda accidents [5,21]. We considered that the prevalence of boda-boda accidents in Gulu Municipality was 24.1%. With a margin of error of 0.05, we estimated a sample size of 201 boda-boda drivers to have a power of 80% to detect boda-boda accidents observed in Gulu Municipality. This was considered a representative sample size which was sufficient to obtain generalizable information on boda-boda drivers and accidents in this study population. Substituting in the formula using a formula where the total population is known (600 drivers):


$$n=\frac{N*X}{\left(X+N-1\right)}$$

Where:


X=Zα2*P*(1−P)MOE2

Zα/2 is the critical value of normal distribution at α/2 (confidence level of 95%, α is 0.05 and the critical value is 1.96); MOE = the margin of error; p = the sample proportion; N = the total population size; n = sample population (sample size). The total population of registered boda-boda members was 600, with a 95% confidence level (1.96), an estimated prevalence of boda-boda in the community of 24.1% (0.241), and a margin of error of 0.05. We used 24.1% for calculating boda-boda accidents based on a previous study conducted in Gulu Regional Referral Hospital on boda-boda accidents [5]. The calculated sample size of the study population was:


X=Zα2*P*(1−P)MOE2=1.962x0.241(1−0.241)0.05x0.05=3.8416x0.1829190.0025=0.702701630.0025=281.0806522

$$n=\frac{N*X}{\left(X+N-1\right)}$$

$$n=\frac{600x281.0806522}{\left(881.0806522-1\right)}=\frac{168,648.3913}{880.0806522}=191.628$$

Considering a non-response rate of 5%, n=191.628 + 191.628 x 0.05 = 191.628 + 9.5814 = 201.2094. Therefore, the sample size obtained for this study population was 201.

**The sampling technique:** at the beginning of the study, the research team held pre-study visits and held meetings with leaders of each stage to obtain the number of registered members and make appointments for interviews. The boda-boda stage leaders planned drivers´ schedules for interviews with the research team on the appointed date. On completion of the consenting processes, the research team completed face-to-face questionnaire interviews with drivers in the nearby quiet of each stage. Those who were not part of the interviews or had completed interviews continued to serve clients normally so that operations at the stage went minimally interrupted. The total number of drivers selected from each stage varied depending on the stage´s population size and availability of eligible members at the time of interviews but no more than three drivers were selected from each stage, and this was continued until the total sample size was realized. This restricted number of drivers selected per stage was to ensure that undeviating representation of participants across the four divisions was made. As for the victims of boda-boda accidents, the research team collected data from the Surgical Units of Gulu Hospital. The nurse in charge helped the research team identify victims of boda-boda accidents at the accident and emergency department and surgical wards of the hospital. The team then verified the participants using the selection criteria and performed the consenting process. Thereafter, the team held a face-to-face questionnaire interview with participants, attendants, and in charge of the unit.

**Figure 1:**
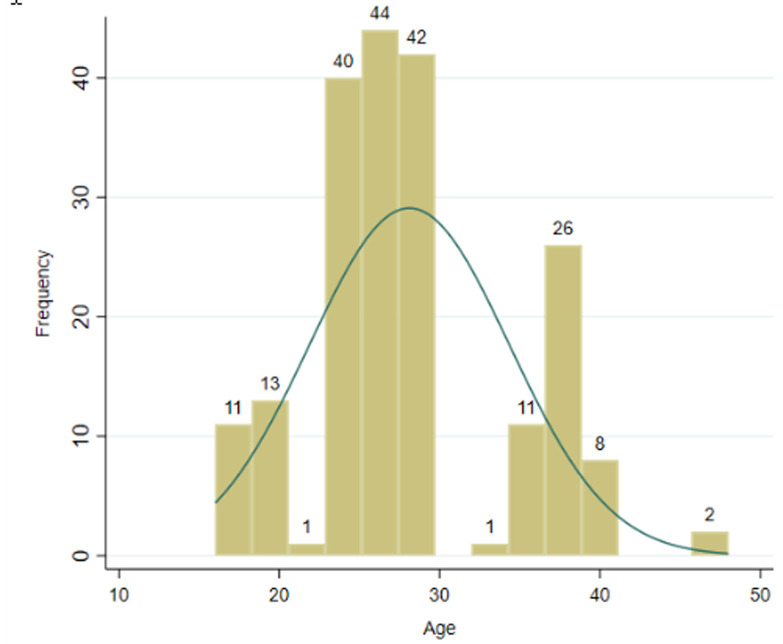
socio-demographic characteristics of boda-boda drivers in Gulu Municipality

**Data collection:** we collected data from drivers and victims of boda-boda accidents using a questionnaire (Figure 1). The nature of the injury was assessed and using the abbreviated injury scale (AIS), injuries were graded as minor, moderate, and severe [26].

**Pre-testing of the questionnaire:** the questionnaire was pre-tested among drivers of motor vehicle taxis in Gulu Municipality to determine consistency, flow, and understanding of the questions. Any unclear questions were noted and reviewed by the research team to ensure clarity, consistency, value, and relevance. Questions that were not relevant or applicable in the situation were modified, replaced, or deleted from the line of questions set in the questionnaire. Any additional questions from the pre-test which were omitted or were overlooked were returned to the final document after the pre-test. The team observed an internal validity and consistency of 72% on questions in the questionnaire (Cronbach´s alpha, p=072) [27].

**Data collection procedures:** we collected data from drivers using a face-to-face questionnaire interview which lasted between 45-60 minutes in the nearby quiet of their workplaces (Figure 1). The questionnaire was designed by the research team and had structured questions (SQ) and unstructured questions (UQ). It had questions on the socio-demographic and personal characteristics of drivers such as age, tribe, religion, stage, division, sex, daily and monthly incomes, occupations, level of education, a time when work starts and ends, house ownership, availability of running water and electricity in the house, marital status, number of children, and ownership of the motorcycle, knowledge on road safety rules and regulations, training in driving motorcycle, valid drivers´ permit, use of helmets and other protective gears and their attitudes towards road use, accidents and how to handle injured colleagues and passengers. The UQs was open-handed and connected to boda-boda drivers´ knowledge and attitudes towards boda-boda use, values in the transport industry, road traffic accidents, and attitudes towards colleagues who frequently cause accidents. In addition, UQs provided the opportunity for drivers to freely express their thoughts and views on the subject matter. Secondly, victims of boda-boda accidents were interviewed at surgical units of Gulu Regional Referral Hospital (hospital data). The staff nurse on duty identified and confirmed trauma cases that had been detained or admitted due to boda-boda accidents in the hospital. We used a questionnaire that collected demographic and personal characteristics of accident victims (age, sex, religion, tribe, occupation, marital status, addresses, division, and level of education), time of the accident, circumstances of the accident, part of the body injured and places where the boda-boda accidents occurred. The research team was Acholi speakers who were trained on the questionnaire and were able to collect the required information from drivers and victims. However, most participants could speak English, the language used in the questionnaire. When a participant was not able to understand a question in English, translation to Acholi was conducted by the research team. Encouragingly, less than 5 participants (less than 2.5%) needed additional translation to Acholi during the interviews.

**Variables:** key variables for this study were boda-boda drivers, their average daily and monthly incomes, accident events, their socio-demographic characteristics for example age, sex, tribe, religion, level of education, occupation, marital status, stages, divisions, addresses, a time when their work begin and end, house ownership status, the availability or absence of running water and electricity in their houses, the number of children, their knowledge on the road safety rules, use of helmets and other protective gears, how to handle injuries in cases they occurred, their families and income, ownership of boda-boda, training in motorcycle operations, and attitudes towards accidents and victims of boda-boda injuries. For victims of boda-boda accidents, we obtained their background information (age, sex, tribe, religion, level of education, occupation, divisions where he/she got the driver, addresses), the time of the accident, where the accident occurred, what they considered was the causes/circumstances of the accidents, the site of injuries and using abbreviated injury scale (AIS), the injury was graded as minor, moderate and severe [26] and entered into the questionnaire.

**Data management:** all data obtained were double-checked and entered in Excel software and later exported to STATA version 14.1 for cleaning and analysis [28]. Data entry was conducted by two research members and reviewed by the principal Investigator. The consistency of the data was cross-checked, and any inconsistencies were resolved by mutual discussions with the team. Key issues such as socio-demographic characteristics and road safety variables for drivers were re-checked for consistency and the Abbreviated Injury Scale (AIS) [26] was used to grade the injuries. Other categorizations were made with the objective of grouping variables to obtain enough participants for comparison between the different groups. For example, in the age category for those injured, there was one 2-year-old child who was injured as most participants were adults (20-29 years and above).

**Data analysis:** to establish factors associated with boda-boda accidents and injuries in Gulu Municipality, we conducted univariate, bivariate and multivariable logistic regression analyses with boda-boda accidents as the outcome variable [29]. All variables for the multivariable logistic regression analyses were obtained from interviews with drivers and victims of the boda-boda accidents. The confounding variables included background characteristics (age, tribe, sex, marital status, level of education, religions, addresses, house ownership, occupation, number of children and income, availability of running water and electricity in the household), ownership of the motorcycle, and experience in driving, use of helmets and other protective wears, training, time of start and end of driving in a day, alcohol and drug use, knowledge, skills, and attitudes of drivers on road safety rules and regulations. We included each of these variables in the analysis anchored on results from previous studies conducted in different parts of Uganda and elsewhere [2,3,5-7,21]. The multivariable logistic regression analysis was also used to compare variables on boda-boda drivers and victims of boda-boda accidents in the four divisions of Gulu Municipality with boda-boda accidents as the outcome variable.

The principles of the model building were followed until we obtained the final statistical model for this study [30]. We used the basic principle of science by choosing the simplest scientific explanation that fits the evidence that we had observed, maximizing the goodness of fit and bearing in mind the clinical relevance of this research. Wald´s tests guided us in the backward elimination method that was used in the model building [31]. The criteria used for selecting variables included in the multivariable logistic model were based on a p-value of 0.1 or less while excluding those with p-values of 0.05 and above. The goodness of fit model was monitored by increasing values of the log-likelihood and p-values of the coefficients [29]. Item non-response was nil for most key variables and very minimal (less than 2%) where it existed. Given that non-response was very minimal, and that we had adjusted for it in the design of this study and model, we did not make any more adjustments in the analysis. Data analysis was conducted using STATA version 14.1 using logit command to fit variables.


$$\text{logit(y)=}\alpha_{1}+\alpha_{2}x+\cdots \alpha_{t}+\cdots (1)$$

Where: t = number of confounder adjustment variable


$$\alpha +2X_{2}+\alpha_{3}X_{3}+\cdots +\alpha_{t}X_{t}=\text{variables for very small strata which were not of interest}$$

The strata were made in those that caused accidents and those that did not in the four divisions of Gulu Municipality (Laroo, Pece, Layibi, and Bardege).


$$\beta_{1}X_{1}+\cdots +\beta_{n}X_{n}=\text{variables of interest and any variable used in the adjustments}$$

For each accident variables.


$$x_{1}\;\cdots \;\cdot {\text{x}}_{\text{n}},x_{n}=\left\{\begin{array}{c} yes(Had\text{ accident})\\ No(Had\text{ no accident}) \end{array}\right.$$

For the pairs of responses.


$$x_{2}=\left\{\begin{array}{c} 1\text{ pair 2}\\ 0\text{ else} \end{array}\right.,x_{3}=\left\{\begin{array}{c} 1\text{ pair 3}\\ 0\text{ else} \end{array}\right.,\cdots \;\cdots \;\cdot \;\cdot {\text{ x}}_{\text{t}}=\left\{\begin{array}{c} 1\text{ pair t}\\ 0\text{ else} \end{array}\right.$$

Using the statistical model-building methods mentioned above, four statistical models were developed. The first model examined the relationship between boda-boda accidents and income levels of drivers in the four divisions of Gulu Municipality. The second model was the associated factors with accidents for example age and work experience. The third model had levels of education, tribe, religion, marital status, and the number of children of boda-boda drivers. Note that in models two and three, none of the variables featured in any relationship with boda-boda accidents and were considered confounding variables in the model. The fourth model introduced the main occupation of drivers, availability of driving permits, ownership of the motorcycle, and knowledge of road safety rules.

**Figure 2:**
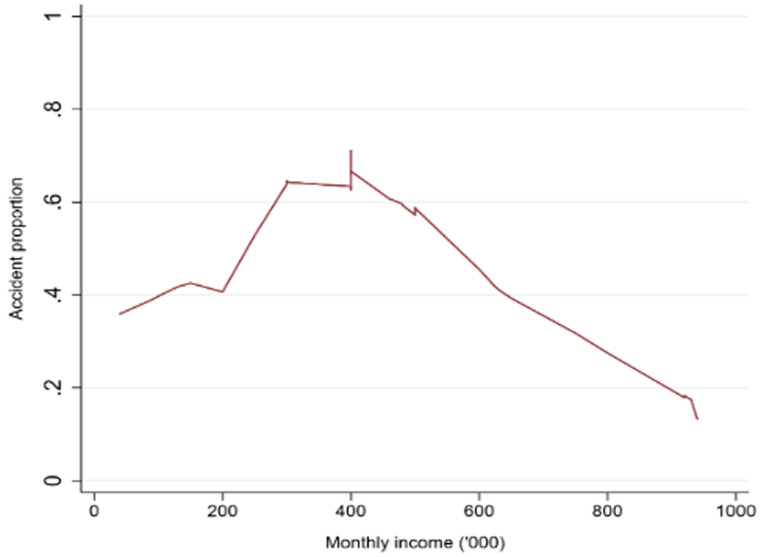
lowess scatterplot between accident proportions and monthly incomes of boda-boda drivers in Gulu Municipality


**Determination on how poor monthly incomes among boda-boda drivers were associated with accidents among drivers in Gulu Municipality:** first, we explored the relationship between boda-boda accidents and monthly incomes using a locally weighted scatterplot smoothing (lowess) (Figure 2). The lowess graph showed a convex relationship between accident proportions and monthly incomes which rose from 0.35 to a maximum of 0.62 at the monthly income of (UGX400,000/=) and then gradually declined to a minimum of 0.15 at the monthly income of UGX940,000/=. At this stage, we subdivided monthly incomes into three terciles for our subsequent analysis. The first tercile ranged from UGX40,000/= to UGX300,000/=, the second from UGX300,000/= to UGX620,000/=, and the third from UGX620,000 to UGX940,000/=. Secondly, we estimated the logistic regression model where the outcome y, occurrence of boda-boda accident was regressed on monthly incomes, divided into the three terciles, and with the second tercile as the omitted reference category (RC). Exp(β_1_) and exp(β_2_) then giving the odds ratios for the poorest and richest terciles compared to the middle tercile respectively. We added covariates (x) to check the sensitivity of our model. The first covariates we added were Gulu Municipal divisions, age, and work experience. Secondly, we added covariates from socio-demographic characteristics (marital status and number of children) and later, tribe, religion, and level of education. Finally, we added covariates about work conditions namely, driving as the main occupation the availability of valid drivers´ permits, and ownership of the boda-boda motorcycle.

$$\text{logit(y=1)=}\alpha \text{=}\beta_{\text{1}}poorest+\beta_{2}Richest+p'x$$

The final analysis showed that the logistic regression model remained stable, and analysis observed that irrespective of adding and removing confounding variables from the model, the model showed that poor monthly incomes were statistically and significantly associated with boda-boda accidents in Gulu Municipality (AoR=0.154, 95%CI: 0.031-0.766; p<0.05) and particularly those from Pece division (AoR=7.290, 95%CI: 2.162-24.580; p<0.001). However, drivers with better incomes more than UGX400,000/= were less likely involved in boda-boda accidents (AoR=0.104, 95%CI: 0.038-0.281; p<0.001).

**Sensitivity analysis and model validation:** we conducted sensitivity analysis and model validation of our findings on the understanding that: 1) all models possess inherent limitations for generalization; 2) there were assumptions made in making causal inferences with the available data; 3) it helped in checking the model fit and performances, especially controlling for confounding variables, for example, the reported information on accidents by boda-boda drivers [32]. This was because we had used reported observations on drivers such as knowledge and socio-demographic characteristics as exposure variables and boda-boda accidents as the outcome variable. The multivariable logistic regression models adjusted for confounders and this was used to compare boda-boda accidents and no accidents in the four divisions of Gulu Municipality [32,33]. In addition, Chi-square tests were used to compare reported and observed injuries by boda-boda drivers and victims of boda-boda accidents in the hospital dataset and we found that the two datasets were comparable (X^2^=6.4035, p=0.094); an indication that the observed distribution of participants in the two datasets was likely due to chance; a reflection of the goodness of fit between reported and observed variables [34].

To ascertain whether there were any misclassifications of data due to social desirability biases [35], a second sensitivity analysis was conducted to assess how robust our self-reported accidents were comparable to hospital data on boda-boda injuries observed in the same period. It had been observed that the reported number of accidents among boda-boda drivers were more than those in the Gulu Hospital dataset and we used the covariate (municipal divisions) where the accidents occurred in the two study groups to impute the missing injuries observed in the hospital data [34]. These missing data may not be surprising as some boda-boda accidents that occurred in Gulu Municipality were managed in clinics and hospitals other than Gulu Regional Referral Hospital where the hospital datasets were collected. We used the Chi-square test on reported variables for the unaccounted-for information on the hospital data to impute the missing boda-boda accident and injury data. We found that the two datasets were comparable as shown by the Chi-square test (X^2^) =6.4035; p=0.092.

**Ethical approval:** this study was approved by a local IRB (Lacor Hospital Institutional Research Ethics Committee (IREC)) and the Uganda National Council of Science and Technology (UNCST). Written informed consent was obtained from each selected participant before administering the questionnaire which contained details on the purpose, objectives, and benefits of the research to road users and policymakers. In addition, the research team assured participants that confidentiality of their information and the security of their data would be maintained. Only the principal investigator had access to their personal information that was de-identified, anonymized, and remained confidential.

## Results

Overall, the questionnaire response rate for this study was 200/201(99.5%).

**Figure 3:**
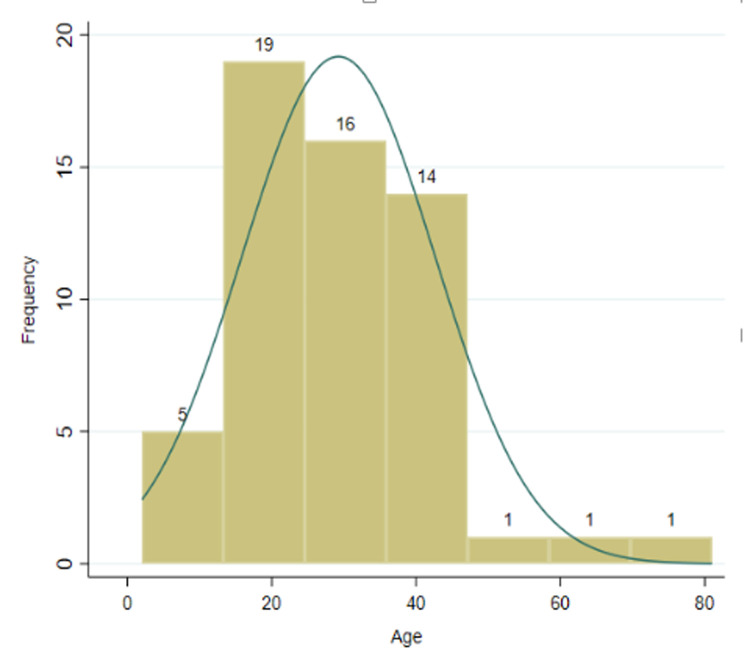
the socio-demographic characteristics of victims of boda-boda accidents in Gulu Municipality

**Socio-demographic characteristics of boda-boda drivers and victims of accidents:** this study showed that socio-demographic characteristics of boda-boda drivers in Gulu Municipality were young adults, consistent with other studies conducted in Uganda [2,3,5-7,21]. Drivers and accidents victims were uniformly distributed across the four divisions of Gulu Municipality (Laroo, Pece, Bardege, and Layibi). Findings reflect the general Ugandan demographic profiles over the years consisting mainly of young people in the age group of 20-30 years [4]. In this current study, most boda-boda drivers were males 197 (98.5%), in the age-group of 20-29 years 127 (63.5%) (Figure 1), Catholics 119 (59.5%), single 118 (59.0%), certificate holders of education 83 (41.5%), from Laroo division 55 (27.5%), had a child 104 (52.0%), Acholi by tribe 181 (90.5%), occupation (boda-boda driving) 183 (91.5%), did not own a house 143 (71.5%), owned a helmet 157 (78.5%), had no electricity 135 (68.0%) or running water in their houses 137 (68.50%) (Table 1). Figure 2 is a lowess scatterplot showing a convex curve between accident proportions and monthly incomes of boda-boda drivers. A direct relationship between accident proportion (from a minimum of 0.35 to a maximum of 0.65) with (a minimum monthly income from UGX<40,000/= to a maximum at UGX400,000/= [equivalent to USD$100]). Thereafter, an inverse relationship between accident proportions (0.65 to 0.15) and monthly incomes (UGX400,000/= to UGX940,000/=) [equivalent to USD$250]). Figure 3 is a histogram describing the age distribution of victims of boda-boda accidents. The majority were 20 years old with a mean age of 29.2 years SD+13.38, a minimum age of 2 years, and a maximum of 81 years.

**Table 1:** socio-demographic characteristics of boda-boda drivers in Gulu Municipality

Ages (years)	Frequency	Percent (%)
10-19	24	12.0
20-29	127	63.5
30-39	47	23.5
40-49	2	1.0
**Sex**		
Male	197	98.5
Female	3	1.5
**Religion**		
Catholics	119	59.5
Protestants	53	26.5
Muslims	7	3.5
Evangelicals	16	8.0
Traditionalists	4	2.0
**Marital status**		
Married	60	30.0
Single	118	59.0
Separated/divorced	21	10.5
Widowed	1	0.5
**The highest level of education attained**		
No formal education	4	2.0
Primary	3	1.5
Secondary	10	5.0
Post-secondary (certificates)	83	41.5
Diplomas	22	11.0
Graduates	27	13.5
Postgraduates	46	23.0
**Occupations**		
Boda-boda driving	183	91.5
Students	7	3.5
Civil servants	3	1.5
Teachers	3	1.5
Farmers	2	1.0
Business	2	1.0
**Tribes**		
Acholi	181	90.5
Baganda	2	1.0
Lango	14	7.0
Madi	1	0.5
Lugbara	1	0.5
Basoga	1	0.5
**Divisions of the Gulu Municipality**		
Laroo	55	27.5
Pece	53	26.5
Bardege	52	26.0
Layibi	40	20.0
There is electricity in my house	64	32.0
There is no electricity in my house	135	68.0
My monthly payment for electricity is less than UGX20,000/=	34	17.0
My monthly payment for electricity is more than UGX20,000/=	30	15.0
There is running water in my house	63	31.5
There is no running water in my house	137	68.5
My monthly pay for water is less than UGX20,000/=	39	19.5
My monthly pay for water is more than UGX20,000/=	9	4.5
I own a house	57	28.5
I have a child	104	52.0
I own my helmet	157	78.5

**The multivariable logistic regression analysis on factors associated with accidents among boda-boda drivers in Gulu Municipality:** in Table 2, using model 4 of the regression analysis, boda-boda drivers with the poorest monthly incomes had AoR=0.154 (95%CI: 0.031-0.766; p<0.05), and from Pece division AoR=7.290 (95%CI: 2.162-24.580; p<0.01) were the most likely involved in accidents while those with the highest monthly incomes AoR=0.104 (95%CI: 0.038-0.281; p<0.01) were least likely involved in accidents at 95% confidence levels. It was determined that the factors associated with boda-boda accidents and injuries were poor monthly incomes (AoR=0.154, 95%CI: 0.031-0.766; p<0.05) and boda-boda drivers from the Pece division (AoR=7.290, 95%CI: 2.162-24.580; p<0.001). Higher monthly incomes more than UGX400,000/= were inversely related to boda-boda accident proportions (AoR=0.146, 95%CI: 0.060-0.353; p<0.001) (Table 1). In Table 2, most victims of boda-boda accidents were passengers and pedestrians whose addresses were from villages outside Gulu Municipality 47.4% (95%CI: 34.5%-60.6%), the main reason for the accidents were over-speeding 36.8% (95%CI: 25.1%-50.4%), and sustained minor injuries 52.6% (95%CI: 39.4%-65.5%), were the secondary level of education 52.6% (95%CI: 39.4%-65.5%), the limbs were most injured 64.9% (95%CI: 51.4%-76.4%), wounds were the commonest injury 70.2% (95%CI: 56.7%-80.9%), peasant farmers 35.1% (95%CI: 23.6%-48.6%), Catholics 50.9% (95%CI: 37.7%-63.9%); occurring mainly in the evenings 59.6% (95%CI: 46.1%-71.8%), male drivers 70.2% (95%CI: 56.7%-80.9%) and mean age of 29 years 29.2% (95%CI: 25.7%-32.8%). Table 3 describes the socio-demographic characteristics of victims of boda-boda accidents and injuries. Most victims were passengers and pedestrians from villages outside Gulu Municipality 47.4% (95%CI: 34.5%-60.6%), were males 70.2% (95%CI: 56.7%-80.9%), with a mean age of 29 years 29.2% (95%CI: 25.7%-32.8%), peasant farmers 35.1% (95%CI: 23.6%-48.6%), Catholics 50.9% (95%CI: 37.7%-63.9%), had attained a secondary level of education 52.6% (95%CI: 39.4%-65.5%), most accidents occurred in the evenings 59.6% (95%CI: 46.1%-71.8%), the main reason for the accidents were over-speeding 36.8% (95%CI: 25.1%-50.4%), sustained minor injuries 52.6% (95%CI: 39.4%-65.5%), the limbs were most injured 64.9% (95%CI: 51.4%-76.4%), and wounds were the commonest nature of injury 70.2% (95%CI: 56.7%-80.9%). Table 4 is a regression analysis results for victims of boda-boda injuries in which passengers and pedestrians from villages outside Gulu Municipality (AoR=8.809, 95%CI: 3.190%-24.329%; p<0.01) were the most involved in accidents.

**Table 2:** odds ratios from logit models for boda-boda drivers in Gulu Municipality

Models	(1)	(2)	(3)	(4)
Variables	Had accident	Had accident	Had accident	Had accident
Income tercile: poorest	0.281(0.074-1.062)*	0.204(0.049-0.845)**	0.153(0.033-0.708)**	0.154(0.031-0.766)**
Middle	RC	RC	RC	RC
Highest	0.186(0.098-0.354)***	0.247(0.122-0.499)***	0.146(0.060-0.353)***	0.104 (0.038-0.281)***
Division: Layibi		RC	RC	RC
Bardege		1.889(0.695-5.134)	1.618(0.566-4.626)	1.726(0.603-4.941)
Laroo		3.058(1.074-8.705)**	2.756(0.883-8.605)*	3.164(0.985-10.158)*
Pece		5.502(1.906-15.882)***	5.893(1.871-18.556)***	7.290(2.162-24.580)***
Age (years)		1.040(0.983-1.101)	1.047(0.977-1.122)	1.049(0.977-1.126)
Work experience: 0-4 years		RC	RC	RC
5-9 years		0.883(0.411-1.895)	0.889(0.377-2.099)	0.803(0.327-1.970)
10-14 years		1.367(0.508-3.683)	2.241(0.630-7.977)	1.800(0.460-7.036)
Levels of education: others			1.993(0.230-17.287)	2.056(0.215-19.622)
Certificate			1.217(0.449-3.295)	1.485(0.522-4.224)
Diploma			1.647(0.458-5.927)	1.736(0.453-6.651)
Graduate			3.771(1.120-12.694)**	3.145(0.894-11.062)*
Postgraduate			RC	RC
Tribes: Acholi			RC	RC
Others			0.148(0.028-0.778)**	0.137(0.025-0.751)**
Religion: Catholics			RC	RC
Muslims			0.452(0.055-3.752)	0.858(0.095-7.752)
Protestants			1.179(0.500-2.776)	1.112(0.458-2.699)
Others			1.021(0.241-4.317)	1.041(0.233-4.647)
Marital status: married			RC	RC
Separated/divorced			1.310(0.332-5.173)	1.204(0.276-5.255)
Single			2.169(0.877-5.367)*	2.529(0.974-6.562)*
Number of children			1.044(0.848-1.285)	1.035(0.840-1.274)
Driving, main occupation: yes				RC
Driving the main occupation: no				1.104(0.258-4.731)
Boda-boda has a permit: no				RC
Yes				2.273(0.849-6.090)
Who owns boda-boda? myself				RC
Employer				0.619(0.219-1.752)
Others				0.594(0.184-1.915)
Constant	2.375(1.571-3.590)***	0.283(0.045-1.777)	0.136(0.012-1.514)	0.090(0.007-1.221)*
Observations	193	193	184	183

95% confidence intervals in parentheses; ***p<0.01; **p<0.05; *p<0.1; RC: reference category

**Table 3:** baseline characteristics of victims of boda-boda accidents in Gulu Municipality

	%	95% CI (%)	n
Divisions: Bardege	10.5	4.7, 22.0	6
Laroo	8.8	3.6, 19.9	5
Layibi	12.3	5.8, 24.0	7
Villages from outside Gulu Municipality	47.4	34.5, 60.6	27
Pece	21.1	12.1, 34.0	12
Causes of injury: a vehicle knocked the boda-boda	7.0	2.6, 17.7	4
Bad road/road humps/no headlights on boda-boda	19.3	10.8, 32.0	11
Drunkenness/failure to break boda-boda in the night	14.0	7.0, 26.1	8
Over-speeding boda-boda	36.8	25.1, 50.4	21
Overloading boda-boda	1.8	0.2, 12.1	1
Passenger drunk/sick	10.5	4.7, 22.0	6
Reckless pedestrians	10.5	4.7, 22.0	6
Classification of injuries: severe	10.5	4.7, 22.0	6
Minor	52.6	39.4, 65.5	30
Moderate	36.8	25.1, 50.4	21
Level of education: diploma/graduate	8.8	3.6, 19.9	5
No education	3.5	0.8, 13.5	2
Primary	35.1	23.6, 48.6	20
Secondary	52.6	39.4, 65.5	30
Injured sites: abdomen	3.5	0.8, 13.5	2
Limbs	64.9	51.4, 76.4	37
Multiple sites	31.6	20.6, 45.1	18
Kind of injuries: abdominal and chest injuries	1.8	0.2, 12.1	1
Fractures of limbs	12.3	5.8, 24.0	7
Head injuries	1.8	0.2, 12.1	1
Multiple injuries	14.0	7.0, 26.1	8
Wounds	70.2	56.7, 80.9	40
Occupation: boda-boda driving	17.5	9.5, 30.1	10
Business	8.8	3.6, 19.9	5
Peasant farmer	35.1	23.6, 48.6	20
Soldier	1.8	0.2, 12.1	1
Student	33.3	22.1, 46.9	19
Teacher	3.5	0.8, 13.5	2
Religions: Catholic	50.9	37.7, 63.9	29
Other Christian denominations	21.1	12.1, 34.0	12
Protestant	28.1	17.7, 41.5	16
Time of the accident: day	22.8	13.5, 35.9	13
Evening	59.6	46.1, 71.8	34
Morning	17.5	9.5, 30.1	10
Sex: female	29.8	19.1, 43.3	17
Male	70.2	56.7, 80.9	40
Age1	29.2	25.7, 32.8	

**Table 4:** odds ratios for boda-boda injuries as per addresses in logit models

Models	1		2	
Variables	Classification of injury			
Addresses of victims	Odds ratio	[95% CI]	Odds ratio	[95%CI]
Bardege	0.239	(0.024 - 2.373)	0.239	(0.024 - 2.373)
Pece	1.245	(0.349 - 4.444)	1.245	(0.349 - 4.444)
Laroo	2.951	(0.501-17.394)	2.951	(0.501-17.394)
Layibi	0.193	(0.020 - 1.843)	0.193	(0.020 - 1.843)
**Outside municipality [Reference]**				
Constant cut 1	0.988	(0.477 - 2.050)	0.988	(0.477 - 2.050)
Constant cut 2	8.809***	8(3.190 - 24.329)	8.809***	(3.190-24.329)
Observations		57		57

95% confidence intervals in parentheses; ***p<0.01; **p<0.05; *p<0.1

In Table 5, most boda-boda drivers had many responsibilities, including caring for more than 2 children at any one time (79.5% versus 20.5%). Most drivers were not renting their own houses (30% versus 70%) but lived with relatives or friends (70% versus 30%). Those who rented their own houses settled for the cheapest rooms, which were less than USD$12 a month (98.2% versus 1.7%). Most drivers lived in houses without running water (68.5% versus 31.5%) or electricity (68% versus 32%), perhaps an indication of poor quality of life. On knowledge on road safety measures, there was adequate knowledge (80% versus 20%) and they had the required protective gears which were used regularly (80% versus 20%). The majority had good attitudes towards work (85% versus 15%) and gave support to those who were involved in accidents (85% versus 15%). In addition, they recommended boda-boda as an important transport system for Gulu Municipality because it saves lives, is cheap and affordable, fast, and quick, and can be used to navigate inaccessible areas while providing quality service to their clients. Table 6 shows hospital data and reported accident data were comparable and not significantly different. The hospital data and boda-boda drivers' reports on accidents were subjected to multiple imputation calculations to determine whether the reported accident data was comparable to the observed in Gulu Hospital. It was determined that the two datasets were comparable and there was no statistically significant difference between the two datasets using the Pearson’s Chi-square (X^2^) =6.4035; p=0.094).

**Table 5:** responses of boda-boda drivers on the unstructured questions (UQ)

Responses of drivers	Yes (%)	No (%)
Do you have children?	54.7	45.6
Do have more than two children?	79.5	20.5
Do own a house?	28.5	71.5
Are you renting a house?	30.0	70.0
Is your monthly rent less than UGX50,000/=?	98.3	1.7
If you are not renting, are you living with your friends or relatives?	70.0	30.0
Do you have running water in the house you live in?	31.5	68.5
Do you have electricity in the house you live in?	32.0	68.0
Have you ever undertaken training on road safety rules and measures?	53.5	46.5
Do you know the rules on road safety?	80.0	20.0
Have you ever read the Ugandan government road safety rules?	76.0	24.0
Are Ugandan road safety rules available to boda-boda drivers in your stage?	34.2	65.8
Are there regular programs by Ugandan government to train boda-boda drivers?	60.0	40.0
Do you have your helmet for boda-boda driving?	78.5	21.5
Do you use a helmet each time you drive a boda-boda motorcycle?	77.0	23.0
Do you have other protective wears such gloves for driving boda-boda?	80.0	20.0
Do you agree that boda-boda drivers are the main cause of accidents in Gulu town?	54.5	45.5
Do you agree that boda-boda drivers should first obtain training before starting to drive?	85.0	15.0
Do you support the idea that the police should arrest boda-boda drivers without driving term permits?	40.0	60.0
Do you think the police should arrest boda-boda drivers who have caused accidents?	72.0	28.0
Do you think training drivers on road safety rules will reduce the rampant road accidents?	57.5	42.5
Do you think you have enough knowledge of road safety rules?	85.5	14.4
Would you accept a driver who previously caused an accident to carry your relative?	15.0	85.0
Would you accept a driver who previously caused an accident to carry you?	14.0	86.0
Would you accept a driver who previously caused an accident to carry your trusted client?	14.0	86.0
Would you recommend boda-boda motorcycle as the best mode of transport in Gulu town?	17.5	82.5
Do boda-boda drivers have first-aid kit on their motorcycles?	0.0	100.0
Have you ever seen any emergency drugs with any boda-boda driver in Gulu town?	2.0	98.0
Have you previously recommended drugs for a colleague who was involved in an accident?	93.5	6.5
What are the reasons why you would recommend boda-boda as a good transport system in Gulu town?		
It saves time	41.5	
It's cheap and affordable	19.0	
It's fast and quick	14.5	
It's good at navigating inaccessible areas/terrains	7.0	
It provides quality and good care for its clients	1.5	

**Table 6:** comparison between hospital data and boda-boda drivers' report on accidents

Divisions of Gulu municipality						
Sources of data	Bardege	Pece	Laroo	Layibi	Total	%
Hospital	6(20.0%)	12(40.0%)	5(16.7%)	7(23.3%)	30	100
Boda-boda drivers	27(25.6%)	36(34.6%)	32(30.8%)	9(8.7%)	104	100
Total	33(24.6%)	48(35.8%)	37(27.6%)	16(11.9%)	134	100

Pearson Chi^2^(χ^2^) =6.4035; p=0.094; NB: data for victims of boda-boda accidents from villages outside Gulu municipality were excluded from the imputation calculations

## Discussion

The most significant findings from this study were that boda-boda drivers with poor monthly incomes were more prone to accidents, most victims of accidents were pedestrians and passengers from villages outside Gulu Municipality, drivers were young (Figure 1, Figure 3), and most accidents occurred with drivers from Pece division (Table 1, Table 2, Table 3, Table 4). These findings contrast with previous studies conducted in Gulu and other parts of Uganda where the associated factors to boda-boda accidents were alcohol consumption, lack of training, poor attitudes towards road use, recent changes of motorcycles, lack of driving permit, low work experience, lack and poor use of helmets, lack of protective gears, low level of education, age, long duration of work hours and marital status even after adjusting for confounders [2,3,5-7]. However, the higher prevalence of boda-boda accidents in the evenings in this current study was consistent with previous studies in Uganda [3,5,21]. These findings have implications on what policymakers and implementers could do to address drivers´ behaviours and poor mechanical status of motorcycles used in Gulu Municipality.

Interestingly, some boda-boda motorcycles we observed were in poor mechanical conditions with poor headlamp lights which could have contributed to the higher prevalence of boda-boda accidents and injuries in the evenings when it was dark. It was because of this that authors suggested that stricter enforcement and adherence to traffic rules could improve the situation in Gulu Municipality. Also, the authors argued that regular training of boda-boda drivers in Gulu by Ugandan police and Uganda Red Cross Society (URCS) on road safety rules and regulations were commendable and may have contributed to the adequate knowledge on road safety rules (85.5% versus 14.5%) and appropriate attitudes towards road safety rules and regulations (85% versus 15%) (Table 5). These two organizations did an admirable job as regular training may have in part contributed to the lower prevalence of boda-boda accidents and injuries in Gulu compared to other towns in Uganda [5,9,20,21,25]. Besides, most injuries observed in the current study population were minor as graded using the Abbreviated Injury Scale (AIS) (Table 3). This finding is also supported by a study conducted in Tanzania [36]. Despite all these, there were more boda-boda accidents among drivers who earned less than four hundred thousand shillings per month (Figure 2). This could be explained by behaviours of drivers under economic pressures experienced due to increased costs of living in Gulu Municipality [37,38]. This was supported by recent surveys which concluded that Gulu Municipality was among the top ten fastest growing towns in Uganda and the rate of urbanization was fast [38 - 40].

**Increased costs of living and economic pressures among residents of Gulu Municipality:** a recent survey conducted at the Gulu main market showed that food prices were considerably higher compared to most towns in Uganda [37,38]. Likewise, food in most restaurants was charged between UGX8,000-10,000/= and higher compared to most towns in Uganda at UGX1,500-5,000/=. Thus, the over 200% higher food prices in the Municipality compared to most towns including Kampala was a matter of concern [38,39]. Kampala, the capital city of Uganda with a larger population was expected to have more food demands and economic pressures than Gulu. The analysis showed that this was not the case as Gulu Municipality experienced more food pressures due to overwhelming demands from South Sudan (a neighboring country to the north) where local food production was poorly developed or minimal to meet the demands of their population [39-41]. These overwhelming demands from across the border have implications on enormous economic pressures on residents of Gulu, which were driving the low-income earners (including boda-boda drivers) into deeper economic difficulties.

**Rapidly increasing population in Gulu Municipality:** authors hint that higher food prices in Gulu could be attributable to the rapidly increasing population in Gulu Municipality without a matched food production. Increased number of students at Gulu University (a public university) and other higher institutions of learning for example Unyama National Teachers´ College (NTC), private Universities and academic institutions, for example, Uganda Management Institute (UMI), Gulu Clinical Officers´ Training School (Gulu COTS), University of the Sacred Heart, Cavendish University, and others have played a part [42-45]. In addition, the increased number of tourists, truck drivers plying the Great North Road, and staff of expanded government parastatals; all contributed to a larger population and increased food demands in the municipality [38-43]. This had implications as food production in the region was not matched with the increasing demand in the region and beyond. These authors argue that a more proactive organization of agricultural activities in Uganda could provide the necessary remedy to the shortfalls in food production in Uganda and for the neighboring country to the north. As mentioned above, higher food demands from South Sudan (a country just 100 kilometers) from Gulu Municipality may be another factor taken into consideration as limited farming and food production occur [37,42] and most food supplies were procured in bulk from Gulu, one of the closest urban centers connected to Juba, the capital city of South Sudan by a well-paved road [38-41]. Authors argue that local communities would get adversely affected due to increased demands and supplies of food with the increasing effects of globalization and increased trade across borders. These have been noted and experienced by boda-boda drivers in Gulu where the effect of rapid urbanization, globalization, international trade, supply, and demands knock on their doors every day.

**Few but expensive accommodations in Gulu Municipality:** in further explanation to the economic pressures experienced in Gulu, charges for house rents in Gulu Municipality were higher compared to most towns in Uganda [37,38]. A month´s rent for a single room (permanent structure) in Gulu Municipality was costing more than one hundred thousand shillings (UGX100,000/=). This could give a plausible explanation why only 30% of boda-boda drivers were able to rent as the majority (70%) lived with friends or relatives (Table 5). This contrasted with findings from other Ugandan towns where, as low as twenty thousand shillings (UGX20,000/=) could be sufficient to meet a month´s rent [37]. The number of housing units in Gulu Municipality appeared not to match the increased number of residents in the Municipality. This finding calls for action from urban authorities, private sectors, and the Ugandan Ministry for Housing and Urban Development to support the construction of more housing units in Gulu Municipality. More housing units would support the increasing population and reduce costs of the housing thereby reducing the economic pressures on residents including boda-boda drivers.

**The rapid urbanization of Gulu Municipality:** in the current study, the multivariable logistic regression models showed that most boda-boda accidents occurred with drivers from the Pece division and most victims were pedestrians and passengers from villages outside Gulu Municipality (Table 2,Table 4,Table 6). These authors argue that this may have been a result of behavioral problems among passengers and pedestrians who were not conversant with the rapidly growing Gulu town with major road construction being undertaken particularly in the Pece division. At the time of this study, there were many road humps, roadside ditches, open drainages, bends, corners, and road diversions as more road constructions were being coxswained in the Pece division compared to the other three divisions. Boda-boda passengers and pedestrians from villages outside Gulu Municipality perhaps got distracted or scared and fell off motorcycles during boda-boda drives as new developments frequently emerged in Gulu Municipality during the period (Table 3). However, these authors argue that this would not wholly explain why most boda-boda accidents occurred in the Pece division. Our observations have however, found that of all the divisions in Gulu Municipality, Pece division was the most populous [44,45], had more road-side markets, road-side primary schools and bars, and the main Gulu soccer stadium (Pece war memorial stadium) which regularly hosted games and involved large crowds. In addition, during the current study, large parts of Pece division roads were under major road construction and there were many construction holes and humps along the roads [37]. These two factors we argue may have in part provided some reasonable explanations for the higher prevalence of boda-boda accidents in the Pece division compared to the other three divisions of Gulu Municipality (Table 2, Table 3, Table 4, Table 5, Table 6).

Despite all this information, these authors argue that the poor economic conditions of boda-boda drivers may have played the most part in the increased prevalence of boda-boda accidents in Gulu Municipality (Table 2). This was consistent with a report produced by the European Transport Safety Council (ETSC) in 2007 which observed that little was known about the socio-economic dimensions of road traffic injuries, especially on the commercial motorcycle drivers [45]. The report argued that the preponderance of evidence suggested that traffic injury was associated with a social status were those who were low in social status, sustained traffic injuries more often than those who were high in social status, and that social disparity in risks appeared to apply to all groups of road users and all levels of injury severity [45]. This implied that those groups of the population who were disadvantaged in terms of incomes, education, or quality of their residential areas were also disadvantaged as users of the road transport system by sustaining injury more often than the more advantaged segments of the population [45]. In the current study, most boda-boda drivers who earned poorly, less than four hundred thousand shillings per month were from the Pece division. Most boda-boda accidents occurred in the evenings when drivers tended to over-speed and over-compete for clients to cash in as daily incomes were realized to below (Table 2). Accidents that occurred in the evenings were mainly due to the recklessness of drivers when it was obvious that daily earnings were low, and secondly due to poor mechanical conditions of motorcycles with poor headlamps that could not light in the dark of the evenings. As observed, accidents mainly resulted from over-speeding and risky motorcycle maneuvers as they competed to serve more clients hurriedly in the evening hours. This finding was consistent with previous studies conducted in the Gulu [5,46]. These authors suggest that more studies could be conducted in Gulu Municipality, a town which is rapidly urbanizing, just emerging out of war and rapidly developing to strategize on how to properly regulate this transport industry which will continue to play a major role in the growth and development of Gulu for the future.

**Strengths and limitations:** these were two cross-sectional studies that were conducted at intervals of six months (July and December 2015). They have limitations based on the cross-sectional nature of the study design. A case-control or a cohort study would have produced much more powered results for the observations and outcomes we have reported. However, considering that we conducted the study in the four divisions of Gulu Municipality, the results among the four divisions and stages of the boda-boda drivers were comparable and provided generalizable information which was useful. Also, this current study was conducted on two occasions, making it closer to a longitudinal study, however, we got less than 30% of the boda-boda drivers in their respective states but the results of the first and second studies were nearly the same. The authors found that boda-boda drivers frequently change their stages for many reasons which we were unable to determine. Secondly, the study had a problem selecting drivers who were present and willing to take interviews. This may have caused a selection bias of our study participants. Only boda-boda drivers who were interested and did not have pressing issues were available for interviews, otherwise, boda-boda drivers preferred to do what was more gainful. This was however mitigated by working closely with the stage leaders by ensuring that appointments and confirmation for interviews were made in time and followed prudently. Thirdly, because boda-boda drivers were usually busy serving their clients, on bad days when clients were few, they preferred to return to their duties as quickly as possible even when interviews were already in progress. There were no monetary incentives to participants in this current study as funds for compensating the time spent at interviews were not available. This was however, mitigated by making appointments and receiving confirmations from the stage leaders in advance and ensuring that interviews were conducted quickly and yet exhaustively with the help of the stage leaders.

Fourth, daily and monthly incomes recorded by boda-boda drivers were reported as average incomes and were not verified nor confirmed by any bank statements as most boda-boda drivers operated daily cash transactions. These reported daily/monthly incomes may not necessarily reflect the accurate financial status of each boda-boda driver but were used in calculations to determine its effects on the dependent variable (boda-boda accidents). We mitigated the uncertainties on incomes by getting corroborative information from leaders of stages who knew what each registered member earned daily, and we found that the information presented by the boda-boda drivers was consistent, comparable, and reliable. Fifth, recall bias and social desirability bias from this interviewer-administered questionnaire were also potential limitations for this study. For example, boda-boda drivers may have shillyshally to inform interviewers that they were being affected by other life unsettling issues such as unmet bank loans, undesirable family issues, and personal problems that may have affected their decision in driving boda-boda. These social problems were not explored in-depth but could form a basis for further studies on boda-boda drivers in Gulu Municipality. In addition, there could have been some more confounders that were residual that could have arisen due to misclassification of measured or reported variables on boda-boda drivers and victims of boda-boda accidents. These could include factors for example fatigue at work, a new environment in the newly constructed city, mechanical problems with the motorcycles, poor housing, personal loan obligations, pressures from families, and other personal liabilities. At this stage, we were unable to determine all these challenges. We hope that in the future we could conduct a more comprehensive study on the boda-boda drivers in Gulu Municipality and other cities in Uganda using a validated tool such as the Motorcycle Rider Behaviour Questionnaire (MRBQ) [47].

**Generalizability of information generated:** results from this current study can be generalized to most major urban centers in Uganda especially the border towns in proximity to countries with weak economies for example South Sudan and the Democratic Republic of Congo (DRC). These border towns tend to have many boda-boda drivers, and many road traffic accidents are expected.

**Funding:** this study was partly funded by the University of Oxford under the COOL project in addition to the support from individual authors. Funders were not part of the designs, data collection, and analysis of this data.

## Conclusion

The independent factors associated with road traffic accidents among boda-boda drivers in Gulu Municipality were poor monthly incomes and most accidents occurred in Pece division involving passengers and pedestrians from villages outside Gulu Municipality. Socio-demographic characteristics of drivers for example age, occupation, marital status, motorcycle ownership did not statistically affect the outcome of this study. These findings suggest that obehavioraloural issues need to be investigated among drivers and perhaps one of them could be stress resulting from the tough economic environment experienced by drivers due to the rapid urbanization of Gulu City. Several studies have viewed knowledge on road safety use, alcohol use, lack of driving permit, lack of helmets and other road safety wears, poor attitudes, and fatigue as the major drivers of boda-boda accidents in most settings. The paucity of information on the effects of economic pressures experienced by drivers (especially poor monthly incomes) under enormous economic pressure in a rapidly growing urban environment needs further exploration. These authors suggest the following as recommendations: a more detailed and wide-ranging investigation into the economic conditions of boda-boda drivers in Gulu Municipality be undertaken with a view of conducting in-depth analysis on drivers and accidents. Secondly, there is nea edon to retrain boda-boda drivers on other income-generating activities as some divisions do not generate the required resources to meet their daily needs. Thirdly, the police and other law enforcement agencies should provide continuous education to the public (residents, passengers, and pedestrians) on proper road use considering that many accident victims were passengers and pedestrians from villages outside Gulu Municipality. Law enforcement agencies should remain vigilant and apprehend drivers that get involved in reckless driving. Finally, Motorcycle Riders Behaviour Questionnaire (MRBQ) should be used to conduct comprehensive studies on boda-boda drivers in Gulu Municipality and other municipalities in Uganda to determine behaviours of boda-boda drivers about accidents.

### What is known about this topic

- Boda-boda accidents were the commonest reason for trauma admissions in many hospitals in Uganda;
- The prevalence of boda-boda injuries have been progressively increasing in Uganda;
- Boda-boda injuries were a major cause of early deaths and disabilities in Uganda.

### What this study adds

- Poor monthly incomes and economic hardships among boda-boda drivers were associated with accidents and injuries in Gulu Municipality, Northern Uganda;
- Boda-boda drivers with better monthly incomes had lower risks of accidents and injuries in Gulu Municipality, Northern Uganda;
- Pedestrians and passengers from villages outside Gulu Municipality were more prone to boda-boda accidents and injuries.
